# A gold-catalyzed alkyne-diol cycloisomerization for the synthesis of oxygenated 5,5-spiroketals

**DOI:** 10.3762/bjoc.7.66

**Published:** 2011-05-04

**Authors:** Sami F Tlais, Gregory B Dudley

**Affiliations:** 1 Department of Chemistry and Biochemistry, Florida State University, Tallahassee, FL 32306-4390 USA, Fax: (850) 644-8281

**Keywords:** alkynes, cyclocondensation, cycloisomerization, gold-catalyzed, 5,5-spiroketals

## Abstract

A highly efficient synthesis of oxygenated 5,5-spiroketals was performed towards the synthesis of the cephalosporolides. Gold(I) chloride in methanol induced the cycloisomerization of a protected alkyne triol with concomitant deprotection to give a strategically hydroxylated 5,5-spiroketal, despite the potential for regiochemical complications and elimination to furan. Other late transition metal Lewis acids were less effective. The use of methanol as solvent helped suppress the formation of the undesired furan by-product. This study provides yet another example of the advantages of gold catalysis in the activation of alkyne π-systems.

## Introduction

**Figure 1:**
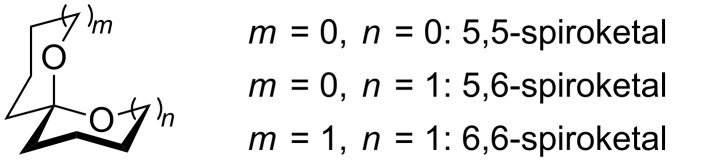
Common spiroketal motifs.

**Figure 2:**
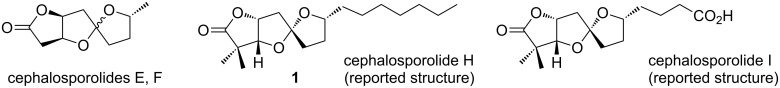
Spiroketal-containing cephalosporolide natural products.

Spiroketals, exemplified by structure shown in Figure 1, are prominent structural features of many biomedically relevant natural and non-natural target structures [1–4]. As such, the synthesis of spiroketals has received considerable attention, with most progress having been made on systems that include at least one six-membered ring [5]. 5,5-Spiroketals (*m*, *n* = 0, Figure 1), particularly oxygenated 5,5-spiroketals such as are found in the cephalosporolides (Figure 2), are the focus of this study.

**Scheme 1:**
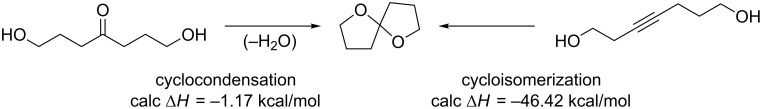
Cyclocondensation vs. cycloisomerization for the synthesis of spiroketals.

A variety of synthetic methods are available for the synthesis of 5,5-spiroketals, including cyclocondensation of ketone diols [6–7], the cycloisomerization of alkyne diols (Scheme 1) [8–16], oxidative spirocyclization of tetrahydrofuryl propanols [17–20], and others. Cyclocondensation of ketone diols is perhaps the most straighforward and the most used method, but the alternative procedures offer specific advantages. For example, the cycloisomerization of alkyne diols is more exothermic (Scheme 1) [21] and atom economical [22], and non-polar alkyne π-bonds are more compatible than ketones (kinetically stable) towards a number of common reaction conditions. Conversely, the use of alkynes in the synthesis of spiroketals introduces regiochemistry concerns as to which of the two alkyne carbons becomes the spiroketal carbon, and the kinetic stability of alkynes must be overcome when alkyne reactivity is desired.

**Scheme 2:**
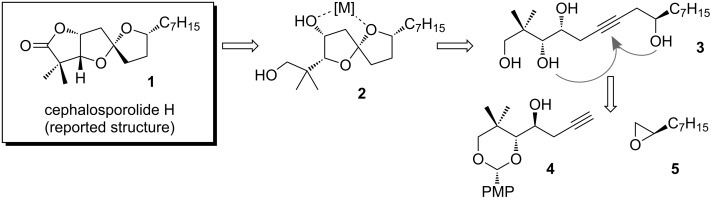
Retrosynthetic analysis of cephalosporolide H.

As an off-shoot of our program devoted to the synthesis of functionalized alkynes by fragmentation reactions [23–27], we became interested in the application of alkyne-diol cycloisomerization to the synthesis of the cephalosporolides and other oxygenated spiroketals. Our retrosynthetic analysis of the reported structure of cephalosporolide H (**1**) is outlined in Scheme 2. We recently demonstrated the use of inter-cycle chelation effects to control the spiroketal stereochemistry [28–29]. However, formation of the requisite oxygenated spiroketals (by cycloisomerization) posed significant challenges that required a focused study.

For this thematic issue on gold catalysis in organic synthesis, we detail here the challenges and considerations involved in the cycloisomerization of alkynes to oxygenated spiroketals and outline our screening of various late transition metal catalysts and conditions that ultimately resulted in the acquisition of our target structures [28]. Gold(I) chloride emerged as the best choice for the desired transformation.

**Scheme 3:**
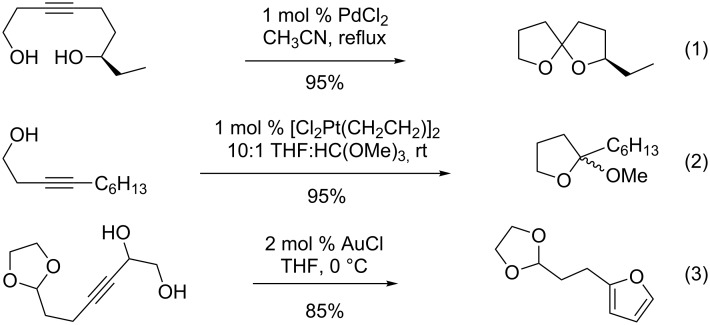
Key precedents for the desired cycloisomerization.

The key precedents for the desired cycloisomerization are shown in Scheme 3, although many methods are available [30–34] and no consensus option has emerged. Utimoto studied the palladium-catalyzed cycloisomerization [8] and reported that a range of spiroketals are available in excellent yield (e.g., Scheme 3, Reaction 1). However, regiochemistry is sometimes difficult to control, and De Brabander later found variability in reaction selectivity using the Utimoto conditions to prepare 6,6-spiroketals. Therefore, he suggested the preferred use of Ziese’s dimer, a platinum catalyst (Scheme 3, Reaction 2), for such cyclizations [9]. In an unrelated study that also bears on the current work, Aponick and co-workers described a gold-catalyzed cyclocondensation of alkyne diols to give substituted furans (Scheme 3, Reaction 3) [35].

**Scheme 4:**
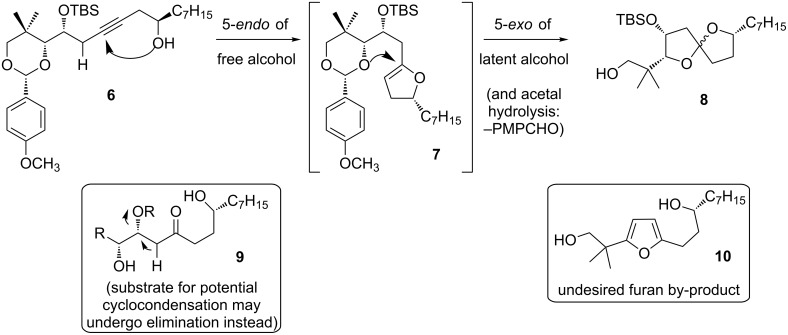
Proposed cycloisomerization with acetal hydrolysis.

Our objective, laid out in Scheme 4, was to initiate cycloisomerization with a 5-*endo*-dig cyclization of the homopropargyl alcohol **6**, followed by 5-*exo*-trig cyclization onto the resulting dihydrofuran, whilst avoiding dehydration to furan **10**. The use of alkyne-diol cycloisomerization instead of ketone-diol cyclocondensation is important for the potential success of this approach, since β-alkoxy ketone **9** (Scheme 4, inset) would be more prone to undesired elimination than homopropargyl ether **6**. We addressed regiochemistry by blocking one of the alcohols as an acetal (the alcohol that otherwise could undergo either 5-*exo* or 6-*endo* cyclization [9,13,36]), thus favoring the initial 5-*endo* cyclization of the other. In this way we aimed to ensure that the desired regioisomer could form, with the expectation of acetal hydrolysis during the course of the reaction. Indeed, attempts to induce spiroketalization after removal of the acetal resulted in complex product mixtures (not shown).

## Results and Discussion

**Scheme 5:**
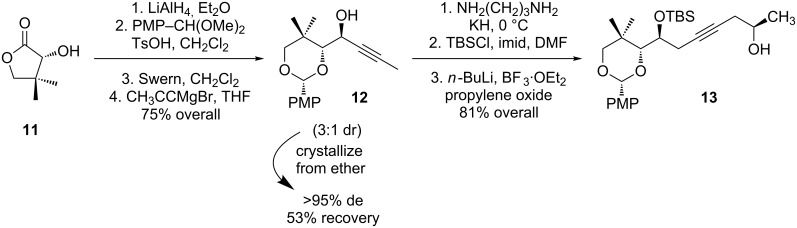
Synthesis of model cyclization substrate **13**.

Initial studies on the cycloisomerization took advantage of chiral propargyl alcohol **12**, which is readily available from pantolactone (**11**, Scheme 5) [37]. An alkyne zipper reaction, protection, and coupling with propylene oxide gave homopropargyl **13**.

Cycloisomerization of **13** to the spiroketal (**14**) was investigated under a variety of conditions, some of which are featured in Table 1. Utimoto’s general conditions as reported (Table 1, entry 1) resulted in decomposition of the substrate, but at room temperature the spiroketal was obtained in modest yield (Table 1, entry 2). Reactions involving Ziese’s dimer were disappointing (Table 1, entry 3), but gold(I) chloride in methylene chloride (cf. Scheme 3, Reaction 3) gave more encouraging results. Other gold catalysts and solvents were screened, with the best results being achieved with a higher catalyst loading of gold(I) chloride in methanol (Table 1, entry 10). The need for higher catalyst loading is tentatively ascribed to some form of instability of the gold catalyst in methanol, as pre-mixing the gold(I) chloride with methanol and aging this mixture prior to adding the substrate results in a less efficient reaction. This is not the first time that we have observed the importance of the order of addition in a gold-catalyzed reaction in a protic solvent [38], but nonetheless we were satisfied with these results for our current study. Furan **16**, which presumably arises by analogy to Aponick’s cyclocondensation, was observed in varying amounts in many cases and was the major product in Table 1, entry 11: The use of methanol as a solvent seems to help suppress formation of the (undesired for our purposes) furan product.

**Table 1:** Spiroketalization using late transition metal salt complexes.

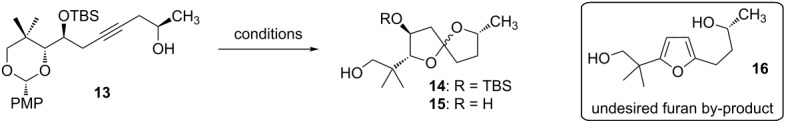			

Entry	Conditions	Major Product	Yield

1	1% PdCl_2_, CH_3_CN, reflux, 1 h	—	—^a^
2	1% PdCl_2_, CH_3_CN, rt, 1.5 h	**14**	43%^b^
3	1% [Cl_2_Pt(CH_2_=CH_2_)]_2_, Et_2_O, rt, then CSA	—	—^a^
4	5% AuCl, CH_2_Cl_2_, rt, 6 h	**14**	36%
5	5% AuCl, PPTS, CH_2_Cl_2_, rt, 14 h	**14**	37%
6	5% AuCl(PPh_3_)_3_, CH_2_Cl_2_, rt	—	—^a^
7	5% AuCl(PPh_3_)_3_, AgSbF_6_, CH_2_Cl_2_, rt, 12 h	—	—^a^
8	5% AuCl_3_, CH_2_Cl_2_, rt, 12 h	—	—^a^
9	5% AuCl, MeOH, rt, 12 h	**15**	35%
10^c^	25% + 25% AuCl, MeOH, rt, 12 h	**15**	68%
11	35% AuCl, MeCN, rt, 4 h	**16**	18%

^a^complex mixture of products was observed, ^b^no increase in yield after a longer reaction time, ^c^a second portion of AuCl (25 mol %) was added after 1 h to achieve full conversion.

**Scheme 6:**
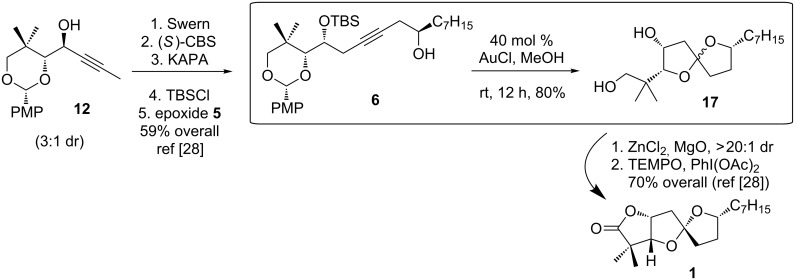
Synthesis of reported structure of cephalosporolide H.

For the synthesis of cephalosporolide H, we prepared homopropargyl alcohol **6** by a two-step inversion of **12**, followed by an alkyne zipper reaction and coupling with nonene oxide (Scheme 6) [28]. Treatment of **6** with 40 mol % gold(I) chloride in methanol resulted in cycloisomerization with simultaneous hydrolysis of the PMP acetal. Meanwhile, cleavage of the silyl ether also occurred under the reaction conditions, and spiroketal diol **17** was isolated in 80% yield as a roughly 1:1 mixture of spiroketal epimers. This mixture of epimers led to a single diastereomer upon chelation with zinc chloride. TEMPO oxidation gave lactone **1**, which corresponds to the reported structure of cephalosporolide H. A more detailed discussion is found in our earlier report [28].

**Scheme 7:**
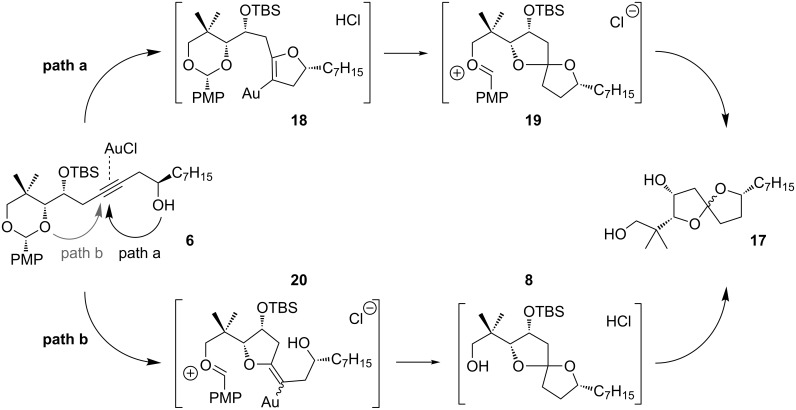
Proposed mechanism.

Two mechanistic alternatives (Scheme 7) are proposed for the conversion of **6** → **17** in methanol. Path a, which corresponds roughly to our original experimental designs, involves initial gold-catalyzed 5-*endo*-dig cyclization to dihydrofuran **18**. Once the regiochemistry is established, any number of condensation pathways would lead to spiroketal **17**. For example, protonation of the enol ether could assist in the opening of the acetal, with simultaneous formation of spiroketal **19**. Any carbenium intermediates could be intercepted reversibly by methanol. The acidity of the gold(I) chloride in methanol mixture is sufficient to hydrolyze the secondary silyl ether group in a separate event: The reaction time was intentionally extended to ensure complete desilylation.

**Scheme 8:**
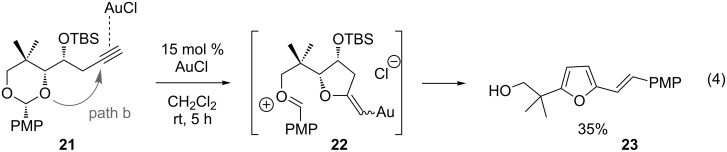
Control experiment for gold-activation of the alkyne.

A second mechanistic alternative, path b, cannot be ruled out at this time. Path b involves gold-activation of the alkyne followed by 5-*exo*-dig nucleophilic attack of the acetal oxygen. Methanolysis of the acetal and spirocyclization would quickly follow. Although this pathway seems unlikely to compete effectively with path a, a control experiment suggests that path b is feasible under certain conditions (Scheme 8, Reaction 4): We subjected terminal alkyne **21** to gold(I) chloride in methylene chloride and observed the formation of furan **23** in low yield, along with other products.

## Conclusion

Gold(I) chloride effectively catalyzed the cycloisomerization of homopropargyl alcohol **6** to spiroketal **17** in good yield, despite the potential for regiochemical complications and elimination to give furan by-products. Other late transition metal Lewis acids were less effective. This study provides yet another example of the advantages of gold catalysis in the activation of alkyne π-systems.

## Experimental

^1^H NMR and ^13^C NMR spectra were recorded in CDCl_3_ as the deuterated solvent. The chemical shifts (δ) are reported in parts per million (ppm) relative to the residual CHCl_3_ peak (7.26 ppm for ^1^H NMR and 77.0 ppm for ^13^C NMR) with TMS as internal standard. The coupling constants (*J*) are reported in hertz (Hz). IR spectra were recorded on a FT-IR spectrometer (100). Mass spectra were recorded either by electron ionization (EI) or fast-atom bombardment (FAB). Yields refer to isolated material judged to be ≥95% pure by ^1^H NMR spectroscopy following silica gel chromatography. All solvents, solutions and liquid reagents were added via syringe. Methanol (MeOH), methylene chloride (CH_2_Cl_2_) and acetonitrile (CH_3_CN) were used without any purification. All reactions were carried out under an inert nitrogen atmosphere unless otherwise stated. Purifications were performed by flash chromatography on silica gel F-254 (230–499 mesh particle size).

Typical procedure for gold-catalyzed spiroketalization: AuCl (8 mg, 0.036 mmol) was added to a solution of **6** (50 mg, 0.091 mmol) in MeOH (5 mL) at room temperature to give a black mixture. After 4 h, the reaction mixture was filtered, mixed with 100 mg of silica gel, and concentrated under reduced pressure. The silica gel admixed with the crude reaction mixture was transferred to a silica gel column and eluted with 15% EtOAc in hexane to afford pure product **17** (23 mg, 80%).


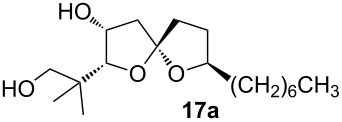


Characterization data for **17a**: ^1^H NMR (600 MHz, CDCl_3_) δ 4.26 (m, 1H), 3.98 (ddd, *J* = 13.1, 9.4, 6.1 Hz, 1H), 3.59 (d, *J* = 3.1 Hz, 1H), 3.51 (d, *J* = 10.9 Hz, 1H), 3.46 (d, *J* = 10.9 Hz, 1H), 2.15 (dd, *J* = 13.4, 4.3 Hz, 1H), 2.10–1.91 (m, 4H), 1.75–1.66 (m, 2H), 1.55–1.47 (m, 1H), 1.40–1.20 (m, 13H), 1.09 (s, 3H), 1.02 (s, 3H), 0.87 (t, *J* = 7.0 Hz, 4H); ^13^C NMR (150 MHz, CDCl_3_) δ 114.1, 91.0, 81.3, 73.3, 71.1, 43.9, 38.0, 37.3, 36.1, 31.8, 30.6, 29.5, 29.2, 26.3, 23.4, 22.6, 20.9, 14.1; IR (Neat): 3280, 2926, 2857, 1461, 1334, 1108; HRMS (ESI^+^): calcd. for C_18_H_34_O_4_Na 337.2354, found: 337.2354.


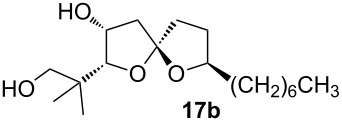


Characterization data for **17b** (obtained as a mixture with **17a**): ^1^H NMR (400 MHz, CDCl_3_) δ 4.39–4.33 (m, 1H), 4.07–3.97 (m, 1H), 3.67 (d, *J* = 10.7 Hz, 1H), 3.65 (d, *J* = 2.9 Hz, 1H), 2.44 (dd, *J* = 14.3, 5.5 Hz, 1H), 2.21–1.98 (m, 5H), 1.54–1.46 (m, 1H), 1.36–1.23 (m, 12H), 1.07 (s, 3H), 1.05 (s, 3H), 0.89 (t, *J* = 6.8 Hz, 3H); ^13^C NMR (100 MHz, CDCl_3_) δ 113.2, 87.9, 78.3, 72.6, 69.7, 69.0, 46.4, 37.5, 36.9, 35.6, 31.8, 30.2, 29.7, 29.3, 25.8, 24.2, 22.7, 21.8, 14.1. IR (Neat): 3280, 2926, 2857, 1461, 1334, 1108; HRMS (ESI^+^): calcd. for C_18_H_34_O_4_Na 337.2354, found: 337.2354.


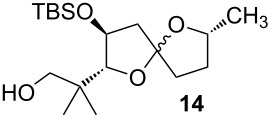


Characterization data for **14**: ^1^H NMR (400 MHz, CDCl_3_) δ 4.49 (m, 1H) [Major], 4.18 (m, 1H) [ Minor], 3.76 (d, *J* = 6.9 Hz, 1H) [Minor], 3.60 (d, *J* = 6.5 Hz, 1H) [Major], 3.49 (m, 1H), 3.39–3.30 (m, 2H), 3.31–2.20 (m, 1H), 2.15–1.86 (m, 4H), 1.68 (m, 1H), 1.43 (m, 1H), 1.29 (d, *J* = 6.1 Hz, 3H) [Major], 1.21 (d, *J* = 6.2 Hz, 3H) [Minor], 0.87 (s, 9H) [Minor], 0.86 (s, 9H) [Major], 0.071 (d, *J* = 1.8 Hz, 6H) [Minor], 0.07(d, *J* = 7.4 Hz, 6H) [Major]; ^13^C NMR (101 MHz, CDCl_3_) δ 113.28, 112.79, 92.23, 89.87, 76.85, 74.43, 72.49, 71.61, 71.49, 71.43, 45.43, 45.15, 37.54, 37.18, 36.75, 36.70, 32.34, 31.77, 25.73, 25.70, 22.68, 22.39, 21.20, 21.04, 20.46, 19.73, 17.78, 17.70, −3.95, −4.01, −4.86, −4.95. HRMS (CI^+^): calcd. for C_18_H_37_O_4_Si 345.2455, found: 345.2455.


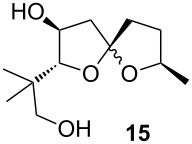


Characterization data for **15**: ^1^H NMR (400 MHz, CDCl_3_) δ 4.57 (dd, *J* = 16.2, 7.1 Hz, 1H), 4.24 (dq, *J* = 6.2, 12.6 Hz, 1H), 4.17–4.06 (m, 2H), 3.86 (d, *J* = 2.6 Hz, 1H), 3.55–3.32 (m, 5H), 3.08 (br s, 1H), 2.95 (br d, *J* = 9.3 Hz, 1H), 2.65 (br s, 1H), 2.57 (br s, 1H), 2.31 (dd, *J* = 12.5, 7.0 Hz, 1H), 2.19–1.88 (m, 9H), 1.76–1.57 (m, 4H), 1.53–1.40 (m, 1H), 1.29 (d, *J* = 6.1 Hz, 3H), 1.22 (d, *J* = 6.2 Hz, 3H), 0.97 (s, 3H), 0.96 (s, 3H), 0.93 (s, 3H), 0.86 (s, 3H); ^13^C NMR (100 MHz, CDCl_3_) δ 114.6, 113.0, 94.5, 91.7, 76.8, 74.8, 72.9, 72.1, 70.9, 70.8, 44.3, 44.1, 37.5, 37.3, 36.9, 34.0, 32.3, 31.6, 22.5, 21.5, 21.3, 20.99, 20.84, 18.8; IR (Neat): 3389, 3005, 2969, 2873, 1461, 1350; HRMS (ESI^+^): calcd. for C_12_H_22_O_4_SiNa 253.1416, found: 253.1413.


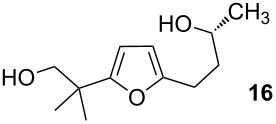


Characterization data for **16**: ^1^H NMR (400 MHz, CDCl_3_) δ 5.97 (d, *J* = 3.1 Hz, 1H), 5.90 (d, *J* = 3.0 Hz, 1H), 3.83 (m, 2H), 3.56 (s, 2H), 2.76–2.62 (m, 2H), 1.77 (m, 2H), 1.23 (d, *J* = 12.8 Hz, 9H); ^13^C NMR (101 MHz, CDCl_3_) δ 114.32, 91.01, 77.14, 73.25, 70.84, 43.98, 38.01, 36.43, 32.16, 23.77, 22.66, 21.01. HRMS (ESI^+^): calcd. for C_12_H_20_O_3_Na 235.1310, found: 235.1315.


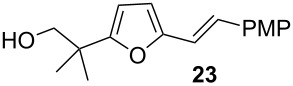


Characterization data for **23**: ^1^H NMR (400 MHz, CDCl_3_) δ 7.40 (d, *J* = 8.9 Hz, 2H), 6.91 (d, *J* = 16.24 Hz, 1H), 6.88 (d, *J* = 8.68 Hz, 2H), 6.71 (d, *J* = 16.24 Hz, 1H), 6.21 (d, *J* = 3.24 Hz, 1H), 6.12 (d, *J* = 3.24 Hz, 1H), 3.82 (s, 3H), 3.64 (d, *J* = 6.56 Hz, 2H), 1.62 (t, *J* = 6.60 Hz, 1H) 1.32 (s, 6H); ^13^C NMR (100 MHz, CDCl_3_) δ 159.9, 159.1, 152.4, 129.9, 127.4, 125.8, 114.7, 114.1, 108.4, 106.9, 71.0, 55.2, 38.5, 23.4. HRMS (CI^+^): calcd. for C_17_H_20_O_3_ 272.1412, found: 272.1421.
